# Herpes Zoster in an Immunocompetent Child Following an Influenza-Like Illness

**DOI:** 10.14740/jmc5400

**Published:** 2026-10-02

**Authors:** Fitri Amalina Md Yusoff, Noorhida Baharudin

**Affiliations:** a Department of Primary Care Medicine, Faculty of Medicine, Universiti Teknologi MARA, Sungai Buloh Campus, 47000 Sungai Buloh, Selangor, Malaysia; b Cardiovascular Advancement and Research Excellence (CARE) Institute, Universiti Teknologi MARA, Sungai Buloh Campus, Jalan Hospital, 47000 Sungai Buloh, Selangor, Malaysia

**Keywords:** Herpes zoster, Varicella zoster virus, Pediatric, Influenza-like illness, Primary care

## Abstract

Herpes zoster (HZ) or shingles is uncommon in children and is traditionally associated with advancing age and immunocompromised states. However, HZ may also occur in immunocompetent children, in whom diagnosis may be overlooked due to its rarity and broad differential diagnosis of pediatric vesicular eruptions. We report the case of an immunocompetent 11-year-old boy with a previous history of natural varicella infection who presented to a primary care clinic with a painful unilateral vesicular rash over the T5–T6 dermatomes. The rash developed 1 week after an influenza-like illness following close contact with a classmate with laboratory-confirmed influenza. A clinical diagnosis of HZ was made based on the characteristic unilateral dermatomal distribution of the rash. The patient was treated with oral acyclovir on day 3 of the rashes onset and recovered completely within 1 week without complications such as secondary infections or postherpetic neuralgia. This case highlights that HZ should be considered in the differential diagnosis of a unilateral dermatomal vesicular rash in children, even in the absence of recognized risk factors such as immunodeficiency. The close temporal association with the preceding influenza-like illness raises the possibility that transient post-viral immune dysregulation may have contributed to the varicella zoster virus reactivation. Although the causality cannot be established from a single case, this observation generates hypothesis that warrants further investigation in future studies. Early clinical recognition and prompt antiviral therapy in the primary care setting may facilitate recovery and contribute to favorable clinical outcomes.

## Introduction

Herpes zoster (HZ) results from the reactivation of latent varicella zoster virus (VZV) and is relatively uncommon in children, with reported incidence rates ranging from approximately 0.2 to 2.2 cases per 1,000 person-years [1]. The incidence increases with advancing age and in immunocompromised individual, reflecting the important role of cell-mediated immunity in maintaining viral latency [1, 2]. Among pediatric populations, HZ is typically associated with underlying immunosuppression, early primary varicella zoster infection, or less commonly, post-varicella vaccination [3, 4].

Although HZ in immunocompetent children has been reported, published pediatric literature predominantly describes complicated or atypical presentations, including herpes zoster ophthalmicus (HZO), optic neuritis, or reactivation following coronavirus disease 2019 (COVID-19) infection or vaccinations [5–7]. Reports of uncomplicated HZ in otherwise healthy children remain relatively limited, particularly those diagnosed and managed entirely in the primary care setting. In Malaysia, published cases have similarly focused on uncommon manifestation such as Ramsay Hunt syndrome [8] or HZ occurring in immunocompromised populations [9].

We report a case of an immunocompetent 11-year-old boy who developed uncomplicated thoracic HZ 1 week after an influenza-like illness. Although a causal relationship cannot be established, the temporal association provides a basis for discussing whether transient immune dysregulation following an acute viral illness may have contributed to VZV reactivation.

## Case Report

An 11-year-old boy with no known medical illness presented to a primary care clinic with a 3-day history of painful vesicular rash over the left thoracic region. One week before the onset of the rash, he experienced influenza-like symptoms after close contact with a classmate who had tested positive for influenza, although he was not tested himself.

The patient had a history of varicella zoster infection at 4 years of age and had not received varicella vaccination. He had no history suggestive of immunodeficiency such as recurrent infection. He did not have chronic medical illness, or long-term medication use. His immunizations were otherwise up to date. Apart from a maternal history of autoimmune skin purpura, there was no significant family history.

On examination, he was afebrile with normal vital signs. Multiple clusters of vesicular lesions on an erythematous base were observed over the left T5–T6 dermatome (Fig. 1a, b). There were no signs of secondary infection, disseminated lesions or rashes elsewhere on his body. Based on the characteristic dermatomal vesicular distribution, a clinical diagnosis of HZ was made.

**Figure 1:**
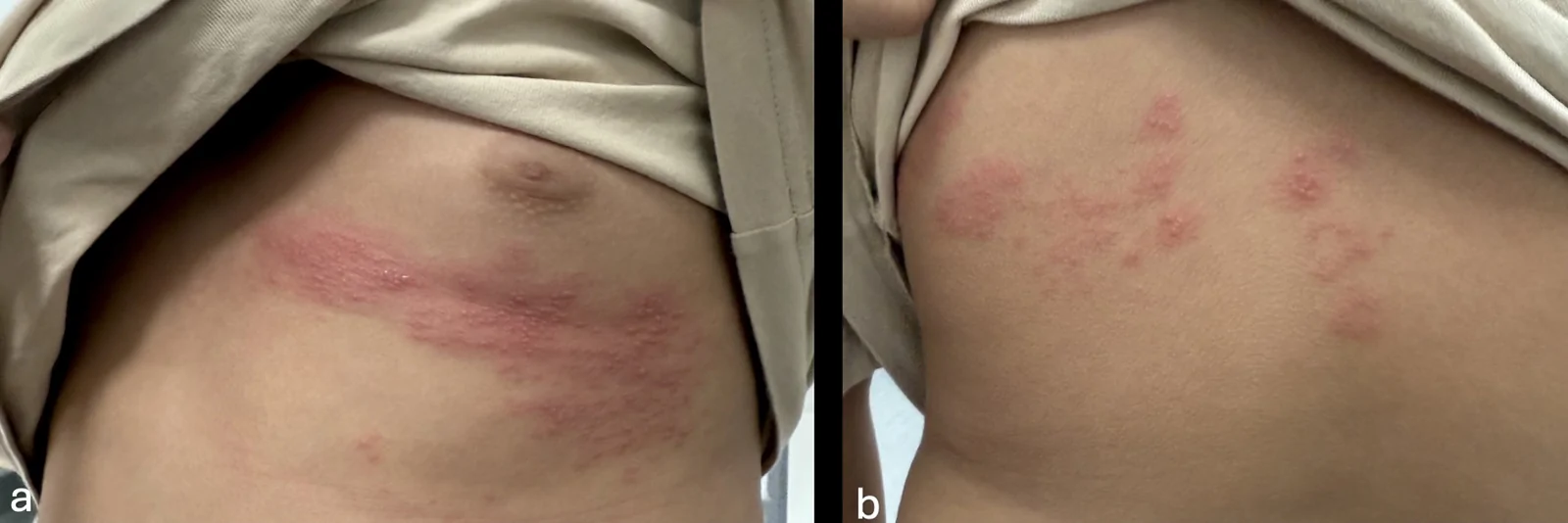
(a) Close-up view of multiple grouped vesicles and erythematous papules distributed over the anterior left T5–T6 dermatomes. (b) Multiple grouped vesicles on an erythematous base over the posterior left T5–T6 dermatomes.

The patient was treated with oral acyclovir and paracetamol for symptomatic relief. He showed good clinical response, with resolution of the lesions and pain within 1 week and no subsequent complications, including postherpetic neuralgia or secondary infections. As there were no clinical features indicating an underlying immunodeficiency, further immunological evaluations were not performed.

## Discussion

HZ or also known as shingles is caused by reactivation of latent VZV [3]. It is uncommon in children, with an incidence rate ranging from 0.2 to 2.2 cases per 1,000 person-years, substantially lower than that observed in adults [1]. Although HZ is classically associated with advancing age and immunocompromised individuals, it can also occur in immunocompetent children without identifiable risk factors [1]. Despite its rarity in childhood, HZ in immunocompetent patients is typically a self-limiting condition with an uncomplicated course and favorable clinical outcome [1]. Our patient represents uncomplicated thoracic HZ in an otherwise healthy 11-year-old boy with a previous history of natural varicella infection and no recognized predisposing factors for HZ. An interesting feature of this case was the appearance of the rashes approximately 1 week after an influenza-like illness. Although the influenza was not confirmed through laboratory investigation, there was a clear epidemiological link through close contact with a classmate with confirmed influenza status. However, the association may have been coincidental as the causal relationship cannot be established.

After primary varicella infection or rarely, following vaccination with a live attenuated VZV strain, the virus establishes lifelong latency in the dorsal root or cranial nerve ganglia [10]. The maintenance of VZV latency relies largely on intact cell-mediated immunity, particularly VZV-specific T-cell responses [2]. Reactivation of the virus is often triggered by a decline in cell-mediated immunity that will lead to its spread along sensory nerves to the skin [2, 10]. Acute viral infections may induce temporary immune dysregulation through several mechanisms, including transient lymphopenia, alterations in cytokine profiles, T-cell exhaustion, and impaired cellular immune responses [2]. Similar mechanisms have been described in the reactivation of other herpesviruses, including herpes simplex virus and Epstein–Barr virus [11]. It is therefore biologically plausible that an acute intercurrent viral illness could temporarily reduce immune control of VZV and facilitate HZ reactivation even in otherwise healthy individuals.

Emerging evidence has recently highlighted the potential role of viral infections as triggers for VZV reactivation. Notably, several observational studies, systematic reviews, and meta-analyses have demonstrated an increased incidence of HZ following COVID-19 infection [12, 13]. Patients with COVID-19 have been reported to have a 2.16-fold increased risk of developing HZ, with almost half of cases occurring within the first week after infection and most presenting as uncomplicated cutaneous disease [12]. Proposed mechanisms include transient suppression of T-cell-mediated immunity, lymphocyte depletion, and disruption of immune surveillance against latent viruses [13]. Although these associations have been most extensively studied in the context of COVID-19, they support the concept that acute viral illnesses may act as triggers for HZ through short-term immune dysregulation. Such observations provide a biologically plausible framework for considering post-viral reactivation of VZV following other viral illnesses [13].

Published pediatric case reports further support this hypothesis. Cases of HZO have been described in immunocompetent children following COVID-19 infection [5], while Miller et al reported vaccine-strain HZ occurring 4 days after acute COVID-19 infection in an immunocompetent toddler previously vaccinated against varicella [6]. Although available evidence remains limited and is largely derived from COVID-19-related reports, they suggest that transient alterations in host immunity associated with acute viral infections may precipitate VZV reactivation in children without underlying immunodeficiency. Taken together, these findings support the broader hypothesis that viral illnesses may act as precipitating factors for HZ in otherwise healthy pediatric populations. Whether influenza or other viral illnesses similarly increase the risk of HZ in children remains uncertain and warrants further research.

The likelihood of an underlying immunodeficiency in this patient was considered low. Apart from a previous history of varicella infection, he had no significant medical history, exhibited normal growth and developmental milestones, and had not experienced recurrent or unusually severe infections. The HZ episode was localized, uncomplicated, and resolved completely following oral acyclovir therapy without postherpetic neuralgia or other sequelae. These findings are consistent with previous literature reporting favorable outcomes among immunocompetent children with HZ [14]. Current evidence also suggests that an isolated, uncomplicated episode of HZ in childhood rarely indicates an occult immunodeficiency, and extensive immunological investigations are generally unnecessary in the absence of warning features such as recurrent disease, disseminated lesions, prolonged course despite antiviral therapy, or severe systemic manifestations [1].

In most pediatric patients, the diagnosis of HZ can be established clinically through a careful history and physical examination [1, 10]. Our patient exhibited the typical features of thoracic HZ, including unilateral pain followed by grouped vesicular lesions confined in dermatomal distribution without crossing the midline. Thoracic dermatomes are commonly affected in children, whereas cranial nerve involvement has been more frequently described in complicated presentations such as HZO and Ramsay Hunt syndrome [1, 5, 8]. Although the clinical features of HZ are often distinctive, the relative rarity of HZ in otherwise healthy children may contribute to delayed recognition or misdiagnosis as other common pediatric skin conditions including herpes simplex infection, impetigo, contact dermatitis, or insect bites [10, 15]. Recognition of the typical dermatomal distribution together with its characteristic clinical features is therefore important to establish early diagnosis, prompt initiation of antiviral therapy, and avoiding unnecessary investigations or delayed treatment.

Early initiation of antiviral therapy particularly within 72 h of rash onset may reduce disease severity and facilitate recovery [3, 16]. Oral acyclovir usually is the first-line treatment for uncomplicated HZs in immunocompetent children [3, 10, 16]. Although the efficacy of antiviral therapy initiated more than 72 h after rash onset has not been systematically evaluated, available evidence suggests no significant difference in pain outcomes between treatment commenced within 48 h and that initiated between 48 and 72 h after rash onset [17]. While evidence regarding treatment beyond this period is limited, some studies suggest that clinical benefit may still be achieved in selected patients, particularly when new lesions continue to appear or complications are present [16].

### Learning points

HZ should remain as differential diagnosis in children presenting with a unilateral vesicular rash, even in the absence of recognized risk factors such as immunodeficiency. Patients with classical presentations can be diagnosed based on history and physical examinations alone, reducing the need for unnecessary investigations in otherwise healthy pediatric patients. Early recognition, diagnosis, and commencement of antiviral therapy even when presentation occurs close to the 72-h window may contribute to favorable outcomes. Although various literature reported acute viral illnesses as potential trigger for VZV reactivation through transient immune dysregulation, further studies are required to confirm this hypothesis.

### Conclusion

This case adds to existing literature describing uncomplicated thoracic HZ following an influenza-like illness in an immunocompetent child. Although causality cannot be established, this observation generates hypothesis that transient post-viral immune dysregulation may contribute to VZV reactivation in susceptible children and warrants further investigation in future studies.

## Data Availability

The authors declare that data supporting this case report are available within the article.

## Abbreviations

HZ: herpes zoster
VZV: varicella zoster virus
COVID-19: coronavirus disease 2019
HZO: herpes zoster ophthalmicus
